# Autophagy Proteins and clinical data reveal the prognosis of polycystic ovary syndrome

**DOI:** 10.1186/s12884-024-06273-w

**Published:** 2024-02-21

**Authors:** Yuanyuan Wu, Jinge Huang, Cai Liu, Fang Wang

**Affiliations:** 1 Gansu University of Chinese Medicine, Lanzhou, 730030 China; 2 Department of Reproductive Medicine, Lanzhou University Second Hospital Lanzhou, Lanzhou, 730030 China

**Keywords:** Polycystic ovary syndrome, Prognostic model, Quantitative proteomics, Autophagy, ARSA, ITGB1, GABARAPL2

## Abstract

**Objective:**

We aimed to investigate the significance of autophagy proteins and their association with clinical data on pregnancy loss in polycystic ovary syndrome (PCOS), while also constructing predictive models.

**Methods:**

This study was a secondary analysis. we collected endometrial samples from 33 patients with polycystic ovary syndrome (PCOS) and 7 patients with successful pregnancy control women at the Reproductive Center of the Second Hospital of Lanzhou University between September 2019 and September 2020. Liquid chromatography tandem mass spectrometry was employed to identify expressed proteins in the endometrium of 40 patients. R was use to identify differential expression proteins(DEPs). Subsequently, Metascape was utilized for Gene Ontology (GO) and Kyoto Encyclopedia of Genes and Genomes (KEGG) enrichment analyses. Multivariate Cox analysis was performed to analyze autophagy proteins associated with reproductive outcomes, while logistic regression was used for analyzing clinical data. Linear correlation analysis was conducted to examine the relationship between autophagy proteins and clinical data. We established prognostic models and constructed the nomograms based on proteome data and clinical data respectively. The performance of the prognostic model was evaluated by the receiver operating characteristic curve (ROC) and decision curve analysis (DCA).

**Results:**

A total of 5331 proteins were identified, with 450 proteins exhibiting significant differential expression between the PCOS and control groups. A prognostic model for autophagy protein was developed based on three autophagy proteins (ARSA, ITGB1, and GABARAPL2). Additionally, another prognostic model for clinical data was established using insulin, TSH, TPOAB, and VD3. Our findings revealed a significant positive correlation between insulin and ARSA (*R* = 0.49), as well as ITGB1 (*R* = 0.3). Conversely, TSH exhibited a negative correlation with both ARSA (-0.33) and ITGB1 (*R* = -0.26).

**Conclusion:**

Our research could effectively predict the occurrence of pregnancy loss in PCOS patients and provide a basis for subsequent research.

**Supplementary Information:**

The online version contains supplementary material available at 10.1186/s12884-024-06273-w.

## Introduction

Polycystic ovary syndrome (PCOS) is a heterogeneous endocrine disorder with an overall prevalence ranging from 6 to 10% according to the diagnostic criteria used [1, 2]. The impact of PCOS on patients is not limited to oligo- or anovulation, as a large number of patients experience poor reproductive outcomes [3–5]. The current research on PCOS mainly focuses on improving ovulatory function, while the mechanisms associated with adverse fertility are rarely mentioned [6, 7]. In other words, PCOS patients still face the risks and challenges of adverse fertility outcomes.

A plethora of data showed that poor reproductive outcomes are associated with endometrial dysfunction [3]. Several studies have found that some clinical and biochemical factors can exert a deleterious effect on endometrium [8–10]. This indicates that clinical and biochemical factors may affect reproductive outcomes.

Autophagy, the primary intracellular degradation system, plays a pivotal role in cellular renovation and homeostasis by recycling waste materials [11]. Previous studies have elucidated the intricate interplay between autophagy, apoptosis, and necrosis. For instance, autophagy can trigger other forms of cell death through selective degradation [12]. Recently, some studies found that autophagic degradation of ferritin leads to ferroptosis due to elevated levels of labile iron and ROS [13, 14]. Some studies have proved that defects in autophagy can lead to follicular development disorders [15, 16]. Furthermore, emerging research has unveiled a link between autophagy and pregnancy loss as it influences immune tolerance at the maternal–fetal interface [17].

This study conducted a secondary analysis of PCOS proteomic and clinical data to investigate the association between autophagy and endometrium, as well as their impact on reproductive outcomes. We analyzed and screened autophagic proteins and biochemical indicators that have a critical impact on the pregnancy outcomes of PCOS. Subsequently, prognostic models were constructed based on characteristic proteins and clinical data, both of which demonstrated robust predictive power. This research significantly contributes to the existing knowledge regarding the relationship between autophagy and pregnancy outcomes in PCOS.

## Materials and methods

### Samples collection

This study is a secondary analysis based on the proteome dataset of endometrium samples obtained from PCOS patients and controls, aged from 21 to 40 years, collected from The Reproductive Center of the Second Hospital of Lanzhou University during the period from September 2019 to September 2020. This dataset included 33 PCOS patients and 7 normal control subjects. The patients who were recruited had to satisfy the Rotterdam criteria meet the following 2–3 items: (1) Oligo-and/or anovulation; (2) Clinical and/or biochemical signs of hyperandrogenism; (3) Polycystic ovaries. The exclusion criteria were as follows: (1) Subjects suffer from hypothyroidism, hyperprolactinemia, adrenal disease, hypertension, and diabetes; (2) hormone-medication and drugs affecting glucose metabolism within the last 3 months. The control group was non-PCOS with successful pregnancy and live birth. They had regular menstrual cycles and normal ovarian morphology via routine ultrasound scans. Informed consent was obtained from all participants before collecting samples. The study was authorized by the Ethics Committee of Lanzhou University Second Hospital (2017A-057).

The endometrial samples were the proliferative endometrium. The endometrial samples were obtained using a pipelle endometrial aspirator and stored at-80℃.

### Clinical and prognosis data collection

Demographic characteristics, including age and BMI, were recorded from outpatient medical records. Serum samples collected during the 2–5 days of menstruation were utilized for the analysis of biochemical indicators, coagulation index, and sex hormones. The analyzed biochemical indicators encompassed serum lipid concentration, fasting plasma glucose levels(FPG), insulin levels, thyroid hormone levels, homocysteine levels, vitamin D3 levels, CA125 levels, and D-dimer. Sex hormones include basal testosterone (T), basal luteinizing hormone (LH), basal follicle-stimulating hormone (FSH), and the anti-mullerian hormone (AMH). The insulin resistance index (IR) is calculated by the HOMA-IR index, which was calculated as fasting plasma glucose (FPG) (mmol/l) × fasting insulin (lU/ml)/22.5, and a value of > 2.6 was considered IR [18]. Endometrial thickness (ET) was examined by ultrasound scanning.

Reproductive outcomes and gestational duration were used as prognostic data, Reproductive outcomes include live birth and adverse fertility. Gestational duration includes the gestational time of live birth and adverse gestational time weeks. Gestational time was estimated in weeks.

Sample preparation and fractionation, data-dependent acquisition (DDA) mass spectrometry, mass spectrometry data analysis, and database search have been described in detail in previous articles [4].

### Obtain the DEPs and the autophagy related proteins

The differential expression protein analysis was based on R package (limma). The screening criteria were |Log_2_fold change (Log_2_Fc)|> 0.585 and adjusted *P* < 0.05 [4]. Autophagy-related proteins (ARPs) derived from the Autophagy Database (http://www.autophagy.lu/).

### The functional enrichment analysis of DEPs

Import DEPs into http://metascape.org/gp/index.html for metascape analysis. Functional and pathway enrichment analysis by Gene Ontology (GO) enrichment analysis and Kyoto Encyclopedia of Genes and Genomes (KEGG) pathway enrichment analysis. Min overlap = 3 and Min Enrichment = 1.5 were the screening conditions. The *P*-value < 0.01 was considered significant.

### Identification of candidate autophagy proteins

We overlapped the ARPs and endometriosis-related proteins. Univariate Cox regression analysis was used to identify the proteins related to pregnancy outcomes. To further identify more reliable autophagy proteins, we conducted LASSO regression algorithm. The “glmnet” package was used to construct the LASSO model with penalty parameter tuning conducted by ten-fold cross-validation. The expressions of candidate autophagy proteins were used to establish a risk model.

### Establishment and evaluation of model

Based on the expressions of candidate autophagy proteins, multivariate Cox regression analysis was used to establish AutoSig Risk Model, Forward and backward method were empolyed for filtering models. The risk score was evaluated by formula as follows: $\mathrm{AutoSig }({\text{PCOS}})={\sum }_{i=1}^{n}coef({Autopro}_{i})*expr({Autopro}_{i})$. AutoSig (PCOS) represents a prognostic risk score, $coef({Autopro}_{i})$ represents the risk coefficient of ith prognostic autophagy protein. $expr({Autopro}_{i})$ is the expression level of the ith prognostic autophagy protein for the patient. The PCOS samples were separated into high-risk and low-risk by the risk score cutoff value (median risk score). Kaplan–Meier method was used to estimate the reproductive outcomes of different groups in R package (survival and survminer). At the same time, Logistic regression was performed for clinical data. The outcome variable was the presence or absence of a live birth. Similarly, forward and backward methods were used for filtering models. We obtained a CliSig Risk model formula as follows: ${\text{P}}=1/\left(1+\mathrm{exp }\left(- \left(\beta +{\beta }_{1}*{x}_{1}+{\beta }_{2}*{x}_{2 }+{\beta }_{3}*{x}_{3}+{\beta }_{4}*{x}_{4}\right)\right)\right)$

The ROC curves were evaluated for the AutoSig Risk Model and CliSig Risk Model. The decision curve analysis (DCA) curves was performed to assess the net benefits with the Risk Model.

### Statistical analysis

The StataSE 15.0 software was used to calculate clinical data. The proteomic data were analyzed by R software. The results were shown as mean ± standard deviation (SD) or median (interquartile range) according to the normal disttribution assumption. The binary logistic regression model was used to develop a CliSig Risk Model, the Cox regression model was used to develop an AutoSig Risk Model. All statistical tests were two-sided, and *P* values of < 0.05 were considered significant.

## Result

### Participant clinical characteristics

Participant clinical characteristics showed significant differences (*P* < 0.05) between PCOS patients and normal controls except for age (*P* = 0.37) (Table 1). The BMI, AMH, FSH, LH, LH/FSH, T, FPG, Insulin, and HOMA-IR of the PCOS group were significantly higher than those of the control group. The ET of the PCOS is thinner than the control. The pregnancy outcomes differences between PCOS and the control group were significant(*P* = 0.043).

**Table 1:** Participant clinical characteristics of patients with PCOS and controls

Variable (n)	PCOS (*n* = 33)	Control (*n* = 7)	*p*-value
Age (year)	25.8 (3.1)	27.0 (2.9)	0.37
BMI (kg/m^2^)	23.9 (21.1, 27.3)	20.8 (19.5, 22.0)	0.03
AMH (ng/mL)	9.0 (4.1)	1.8 (1.1)	< 0.01
FSH (mIU/mL)	7.2 (5.9, 7.9)	5.3 (5.2, 6.3)	0.02
LH (mIU/mL)	11.8 (4.3)	5.3 (0.6)	< 0.01
LH/FSH ratio	1.8 (0.7)	1.0 (0.1)	< 0.01
T (ng/dL)	42.1 (17.6)	24.7 (11.4)	0.02
FPG (mmol/L)	5.2 (0.5)	4.5 (0.4)	< 0.01
Insulin (mIU/mL)	16.2 (9.77, 25.84)	7.34 (6.49, 12)	0.015
HOMA-IR	3.89 (2.35, 6.29)	1.74 (1.29, 2.16)	0.003
ET (mm)	4.0 (1.4)	9.6 (0.8)	< 0.01
Live birth (%)	20(60.6)	7(100)	0.043
Adverse gestation (%)	13(39.4)	0(0)	

*BMI* Body mass index, *AMH* Anti-mullerian hormone, *FSH* Follicle-stimulating hormone, *LH* Luteinizing hormone, *T* Testosterone, *FPG* Fasting plasma glucose, *FINS* Fasting insulin, *HOMA-IR* Homeostasis model assessment of insulin resistance, *ET* Endometrial thickness

*p* < .05 was considered statistically significant

### Endometrial proteomic analysis and differential expression protein analysis

**Fig. 1:**
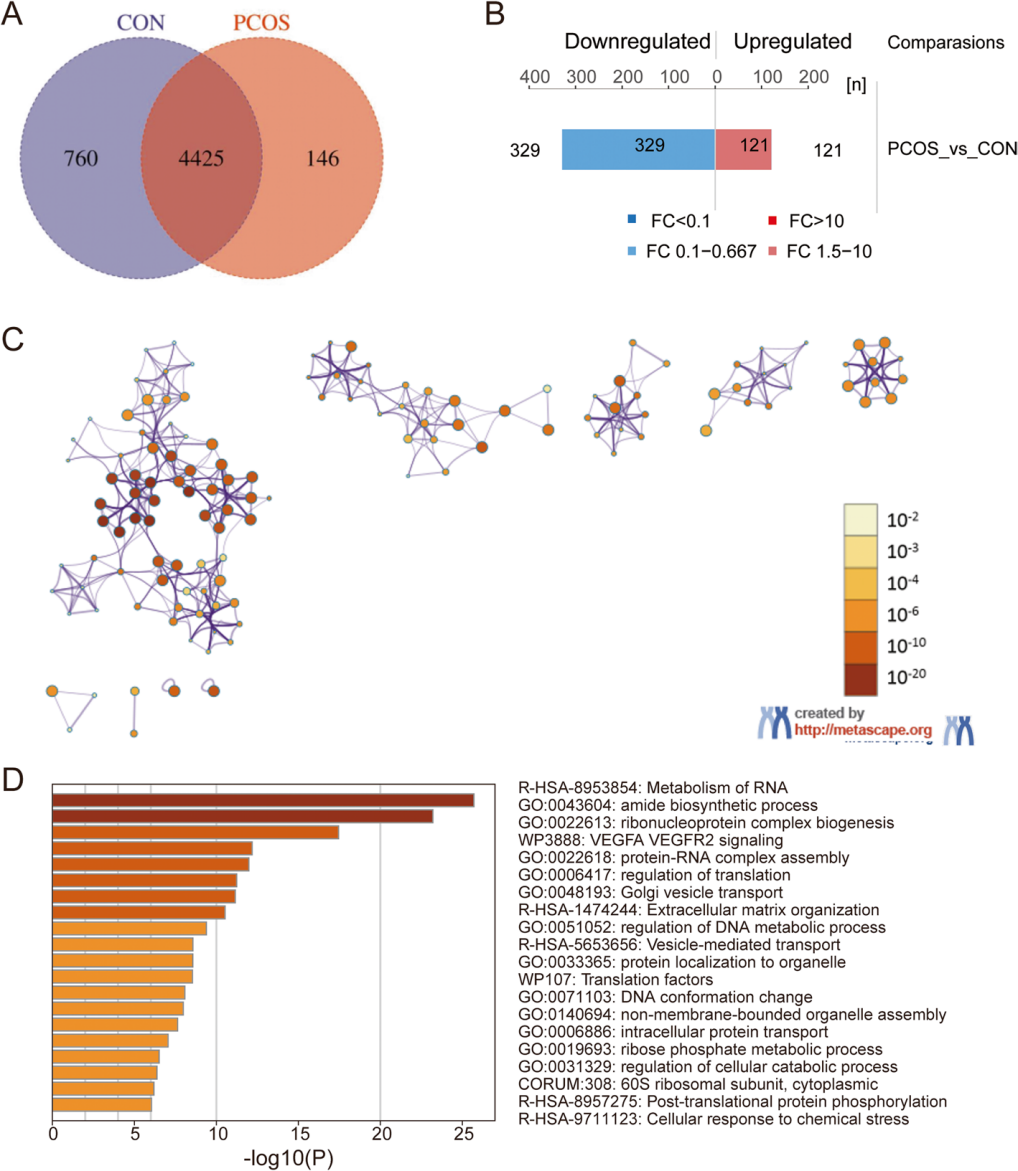
DEPs and Metascape analysis: **A** Venn plot of inter group samples in DIA data; **B** Histogram of protein quantitative difference results; **C** Enriched ontology clusters colored by *p*-value. the dark the color, the more statistically significant the node is. **D** Enriched ontology clusters across studies

A total of 5331 proteins were identified, with 4425 proteins overlapped in PCOS and control group (Fig. 1A). A lot of 450 DEPs (121 up-regulated and 329 down-regulated) were identified. (Fig. 1B, Supplementary file 1).

### The functional enrichment analysis of DEPs

Metascape revealed biological processes containing amide biosynthetic process, ribonucleoprotein complex biogenesis, regulation of cellular macromolecule biosynthetic process, and regulation of DNA metabolic process. The significant biological pathways were the metabolism of RNA, VEGFA-VEGFR2 signaling pathway, and Extracellular matrix organization (Fig. 1C-D, Supplementary file 2). These results indicated that autophagy was greatly involved in the pathogenesis and prognosis of PCOS.

### Identification of candidate autophagy proteins

**Fig. 2:**
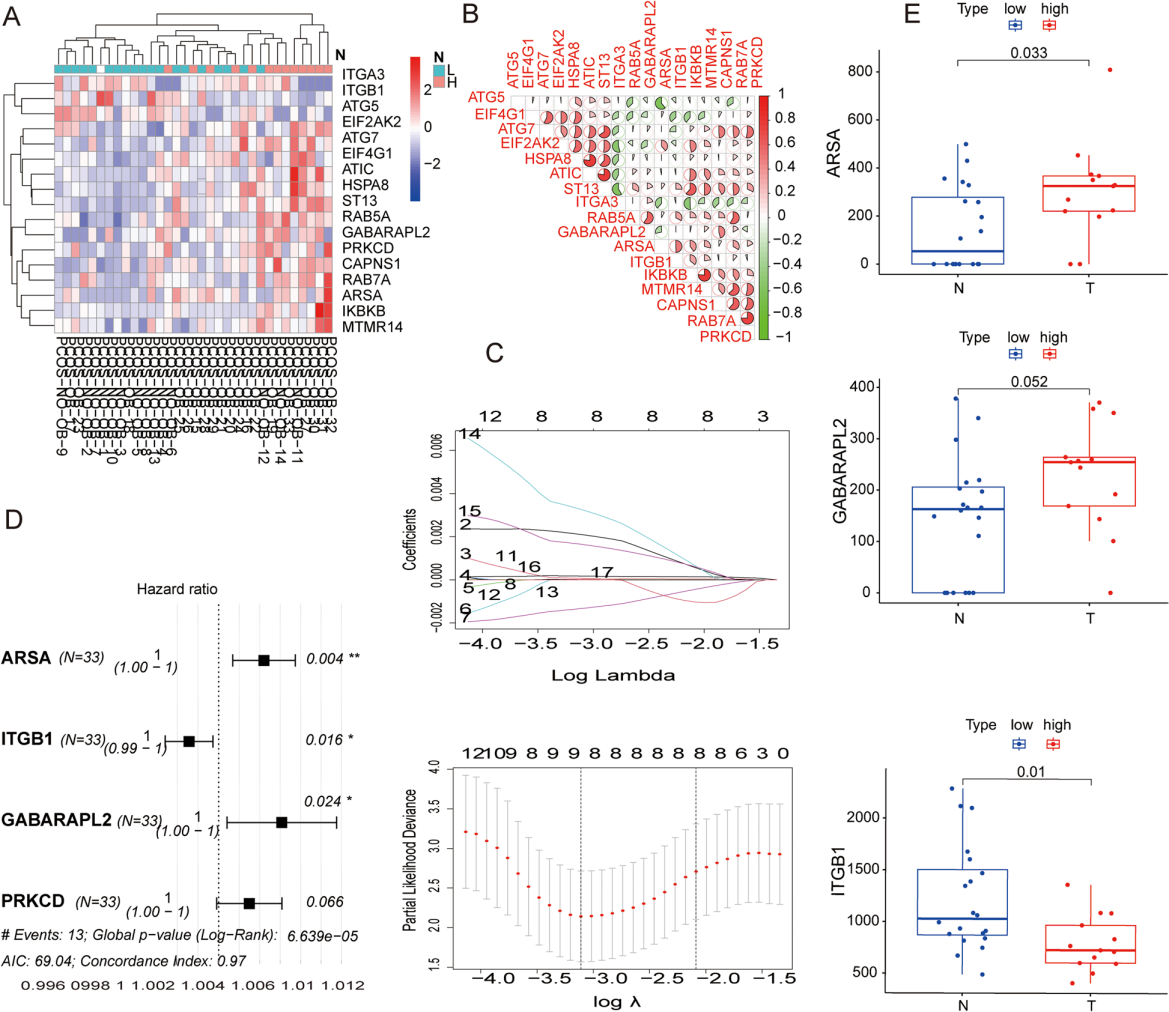
Variable screening of autophagy proteins: **A** Heat map of autophagy proteins related to PCOS pregnancy outcomes; **B** Correlation analysis of pregnancy outcome related proteins; **C** Lasso regression analysis; **D** Forest plots of four autophagy proteins identified by multivariate Cox regression analysis; **E** Expression differences of three autophagy proteins used for modeling in high-risk and low-risk groups

Two hundred thirty two ARPs derived from the Autophagy Database (Supplementary file 3). 83 overlapping proteins were obtained by intersecting ARPs with endometriosis-related proteins. 17 autophagy proteins were significantly correlated with fertility outcomes through univariate Cox regression analysis. To clarify the regulatory relationship between Autophagy proteins related to reproductive outcomes in PCOS, we conducted a correlation analysis using R packets (Fig. 2A-B). Lasso regression analysis was performed to ultimately screen 8 prognostic related autophagy proteins (Fig. 2C). 8 prognostic related autophagy proteins were ARSA, EIF4G1, IKBKB, ITGB1, HSPA8, ATIC, GABARAPL2, PRKCD. 8 candidate proteins selected as for subsequent analysis and construction of risk model.

### Establishment and evaluation of the risk prognostic model

**Fig. 3:**
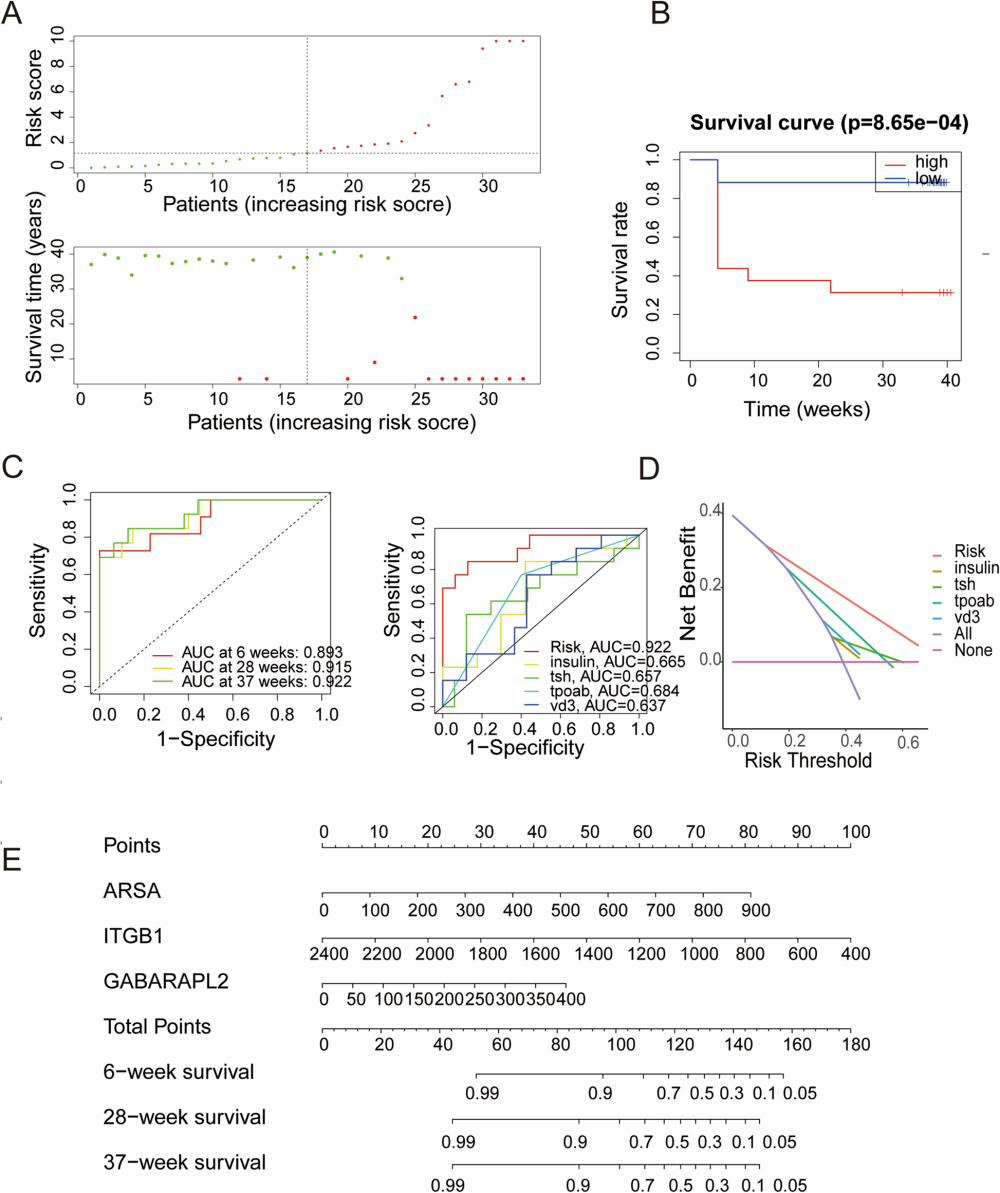
Establishment and evaluation of the AutoSig risk prognostic model: **A** Risk and survival status of PCOS under different risk scores; **B** Survival analysis between high and low-risk groups; **C** ROC of the AutoSig risk model at weeks 6, 28, 37; **D** Decision curve analysis for the AutoSig Risk model; **E** Nomogram to estimate the probability of pregnancy outcome of PCOS use autophagy proteins

Multivariate Cox regression analysis was performed to ultimately screen 3 prognostic related autophagic proteins (ARSA, ITGB1, GABARAPL2) (Fig. 2D). The risk score calculation formula can be obtained: $\mathrm{AutoSig }\left({\text{PCOS}}\right)={0.004908}^{*}\mathrm{expr }\left({\text{ARSA}}\right)+{\left(-0.00272\right)}^{*}\mathrm{expr }\left({\text{ITGB}}1\right)+{0.00628}^{*}\mathrm{expr }\left({\text{GABARAPL}}2\right).$ we found that ARSA and GABARAPL2 had the positive coefficient, suggesting they might be risk factors for a poor prognosis, while ITGB1 had a negative coefficient which indicated it could be a protective factor for live birth. Then, we could use the median risk score to divide the PCOS subjects into high and low-risk groups (Fig. 3A). The differences between the three proteins in high and low-risk groups are plotted in Fig. 2E. Survival analysis showed that the outcomes of pregnancy of low-risk group were consistently better than high-risk group (Fig. 3B). With the increase of risk score, the status of pregnancy decreases significantly. The AutoSig Risk model that incorporated the above independent predictors was developed and presented as the nomogram (Fig. 3E).

The AutoSig risk model was tested by time-dependent ROC curve analysis. At 6 weeks, the mode had the lowest AUC (0.893), At 28 and 37 weeks, the AUC was 0.915, 0.922 (Fig. 3C). The model’s AUC at 37 weeks was significantly higher than the AUC of Insulin (0.665), TSH(0.657), TPOAB (0.68), and VD3 (0.637) (Fig. 3C). This proved that AutoSig had superior prognostic performance. The decision curve analysis for the nomogram is presented in Fig. 3D. indicating that DCA shows a greater net benefit for the AutoSig model over clinical indexes.

**Fig. 4:**
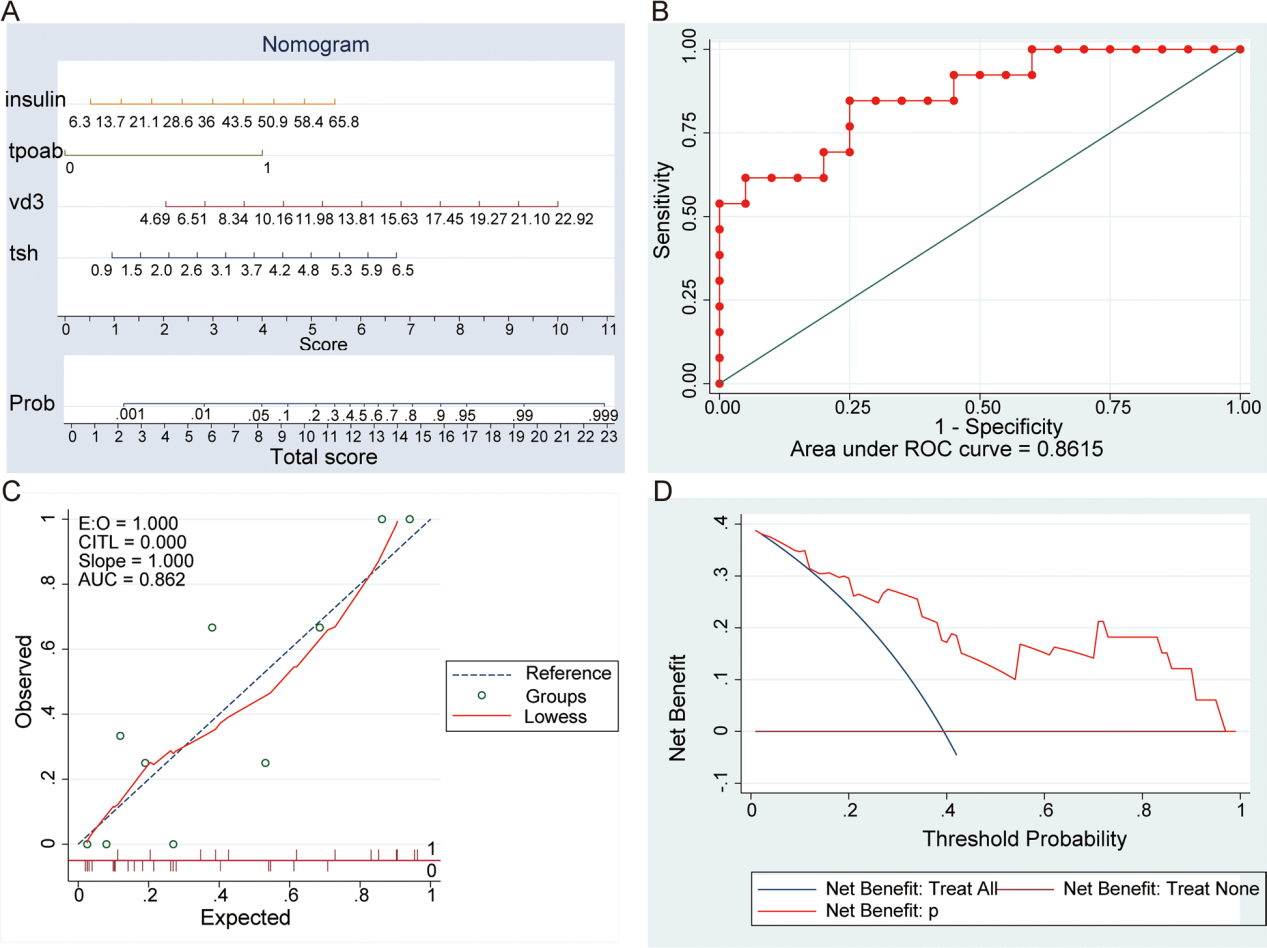
Establishment and evaluation of the CliSig risk model. **A** Nomogram to estimate the probability of pregnancy outcome of pregnancy outcome of PCOS use clinical data; **B** ROC of the CliSig model; **C** The calibration curve of CliSig risk model for predicting pregnancy outcome of PCOS. **D** Decision curve analysis for the CliSig Risk model

27 clinical data as variables (Table 2), Logistic regression was used to ultimately obtain 4 prognostic related clinical data. The clinical data model formula is as follows: ${\text{P}}=1/\left(1+{\text{exp}}\left(-\left({-8.414+0.698}^{*}{{\text{TSH}}+0.292}^{*}{{\text{VD}}3+2.682}^{*}{{\text{TPOAB}}+0.056}^{*}{\text{Insulin}}\right)\right)\right).$ CliSig Risk Model was developed and presented as the nomogram based on the above independent predictors (Fig. 4A). This nomogram had excellent discriminative power with AUC of 0.8615 (Fig. 4B). The Calibration curve was plotted in Fig. 4C. The nomograms were well calibrated, there were no significant differences between the predicted and the observed probability. We did DCA on our prediction model to assess the net benefit that patients could receive (Fig. 4D). The nomogram model has an obvious net benefit for almost all threshold probabilities.

**Table 2:** Analysis of 27 clinical data in pregnancy loss and nonpregnancy loss

Variable (n)	NON-pregnancy loss (*n* = 20)	Pregnancy loss (*n* = 13)	*p*-value
Age(year)			1.00
< 30	18 (90%)	12 (92%)	
> 30	2 (10%)	1 (8%)	
BMI (kg/m^2^)	23.3 (3.4)	25.6 (4.0)	0.09
AMH (ng/mL)	9.4 (4.4)	8.2 (3.5)	0.43
FSH (mIU/mL)	6.7 (1.4)	7.0 (2.0)	0.64
LH (mIU/mL)	12.3 (3.5)	11.2 (5.3)	0.47
LH/FSH ratio	1.9 (0.6)	1.6 (0.8)	0.33
T (ng/dL)	42.0 (11.8)	42.2 (24.5)	0.98
FPG (mmol/L)	5.2 (0.6)	5.2 (0.5)	0.81
Insulin (mIU/mL)	16.3 (9.0)	24.5 (16.1)	0.07
HOMA-IR	3.9 (2.3)	5.7 (4.0)	0.10
ET (mm)	4.3 (1.6)	3.5 (1.1)	0.17
T3(nmol/L)	1.8 (0.3)	1.9 (0.2)	0.45
T4(nmol/L)	109.3 (18.2)	119.8 (15.6)	0.10
FT3 (pmol/L)	5.4 (0.5)	5.5 (0.4)	0.71
FT4 (pmol/L)	15.8 (2.0)	15.3 (1.5)	0.47
TSH (uIU/ml)	2.7 (1.2)	3.2 (1.2)	0.28
thyroglobulin (ng/ml)	14.3 (8.5)	23.8 (21.2)	0.08
ATG-AB(U/ml)			1.00
< 35	19 (95%)	12 (92%)	
> 35	1 (5%)	1 (8%)	
TPO-AB(U/ml)			0.07
< 35	12 (60%)	3 (23%)	
> 35	8 (40%)	10 (77%)	
TC (mmol/L)	4.0 (0.8)	4.1 (0.6)	0.73
TG (mmol/L)	1.4 (0.9)	1.6 (0.9)	0.55
HDL(mmol/L)	1.3 (0.3)	1.3 (0.3)	0.82
LDL(mmol/L)	2.6 (0.7)	2.7 (0.7)	0.71
HCY(umol/L)	12.6 (3.5)	12.4 (6.8)	0.89
VD3(ng/L)	10.0 (4.0)	12.3 (5.1)	0.16
D2 polyme(mg/L)	0.6 (1.2)	0.5 (0.6)	0.79
CA125(U/ml)	15.7 (9.8)	16.5 (9.3)	0.83

*T3* Triiodothyronine, *T4* Thyroxine, *TSH* Thyroid-stimulating hormone, *FT3* Free triiodothyronine, *FT4* Free thyroxine, *ATG-AB* Antithyroglobulin antibody, *TPO-AB* Thyroid peroxidase antibody, *TC* Cholesterol, *TG* Triglyceride, *HDL* High-density lipoprotein, *LDL* Low density Lipoprotein, *HCY* Homocysteine

*p* < .05 was considered statistically significant

### Analysis of linear correlation between clinical data and protein expression

**Fig. 5:**
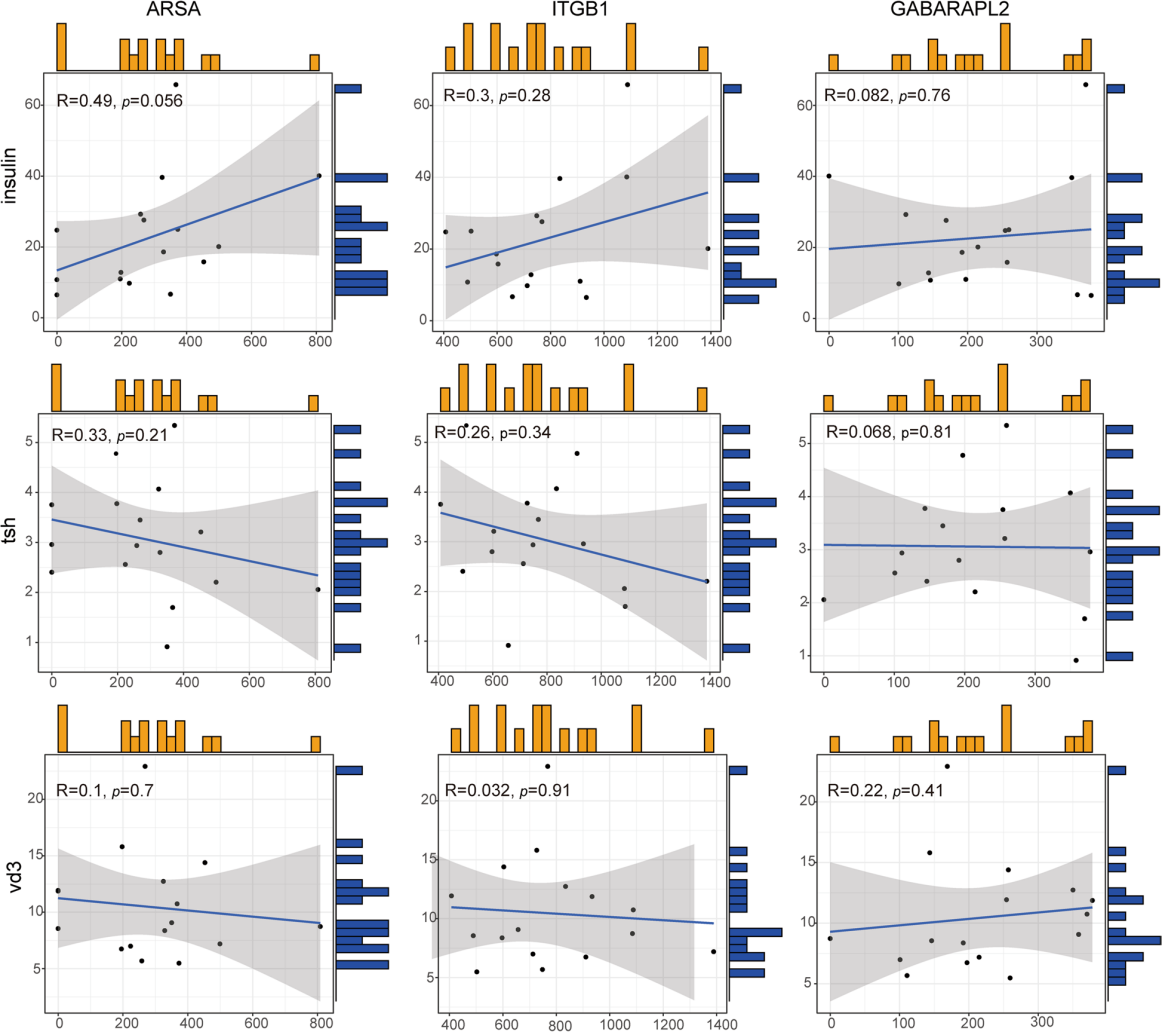
Correlation between clinical data and protein expression

As both models have strong predictive value, we conducted a Pearson correlation analysis between the variables of clinical model and the autophagic protein model. In our study, we found a significant positive correlation between insulin and ARSA (*R* = 0.49), and ITGB1 (*R* = 0.3). TSH has a negative correlation with ARSA (-0.33), and ITGB1 (*R* = -0.26) (Fig. 5).

## Discussion

PCOS is a common gynecological disease characterized by reproductive and metabolic disorders which are related to the occurrence and progression of diseases [19, 20]. The comorbidities of PCOS, including (obesity, metabolic syndrome, hyperinsulinemia or hyperandrogenism), may contribute to pregnancy loss [21]. Obesity and T2DM associated features such as dyslipidemia, oxidative stress, hyperglycemia, hyperinsulinemia could interrupt and compromise autophagy [22]. While poor endometrial receptivity can lead to adverse reproductive outcomes [23]. We could hypothesize that downregulation of autophagy in PCOS patients might lead to poor endometrial receptivity, thereby increasing the incidence of miscarriage. A study using an obese mouse model showed that autophagy was more up-regulated in decidualizing cells of control mice compared to high-fat/high-sugar diet mice [24]. This study attempts to screen the factors most closely related to pregnancy loss in PCOS based on the analysis of PCOS proteomic data and clinical data. Providing a reference for the mechanism research and clinical decision-making.

Molecular functions and pathways could explain the reasons for poor endometrial receptivity in PCOS patients. In our study, the DEPs were shown to be involved in metabolism of RNA pathway. Different types of RNA and RNA-related complexes are recruited to and degraded by autophagy pathway [25]. Lots of studies have demonstrated that inhibitors of autophagosome formation significantly block starvation-induced RNA degradation [26, 27]. The autophagy pathway is damaged, while the metabolism of RNA pathway will be inhibited. This has been positively validated in our research.

In our research, we established two prognostic models based on proteomics and clinical data. After evaluating the models, we found that both models had good predictive performance. The autophagic protein model based on 3 proteins (ARSA, ITGB1, GABARAPL2). Interestingly, our new autophagy proteins model achieved an AUC of 0.922 with only 3 feature proteins, surpassing our previous model which used 5 feature proteins and had an AUC of 0.884. This demonstrates the superiority of our current model. ARSA, ITGB1, GABARAPL2 were rarely studied in PCOS in previous studies. ITGB1 is integrin, which can affect tumor process by regulating angiogenesis, apoptosis, and metastasis [28, 29]. It is widely recognized that ITGB1 involved and promotes the adhesion ability of NCAM1^birgh^ NK cells at the maternal–fetal interface [30–32]. This indicates that there is significant research value in exploring the relationship between ITGB1 and PCOS, and it can also serve as a predictor of pregnancy outcomes in individuals with PCOS. RASA is a lysosomal enzyme that catalyze degradation of sulfatides into galactosylceramides (GalC) [33, 34]. Research has found that the lack or complete absence of ARSA presents metachromatic leukodystrophy which is characterized by the degradation of intellectual function and motor skills and often fatal in early childhood [35–37]. This indicates ARSA may be an important factor in pregnancy loss in PCOS. GABARAPL2 (also called GATE-16) belongs to the GABARAP subfamily of Atg8 proteins [38]. The Atg8 proteins play a key role in the sealing of the isolation membrane which is a vital role in autophagy [39]. This further demonstrates the important significance of autophagy in PCOS.

The clinical data model is based on 4 variables (TSH, VD3, TPOAB, Insulin). The results show that the insulin level is a reliable predictor of pregnancy loss in PCOS. Hyperinsulinemia affects the immune response of the endometrium by decreasing the expression of glycodelin and IGF-binding protein-1 [8], a large number of studies have found that TSH is associated with adverse pregnancy outcomes [40, 41]. Multiple studies have demonstrated the impact of vitamin D on PCOS phenotype and pregnancy loss [42–44]. Research has found a significant correlation between TPO-AB and infertility in patients with PCOS [45].

Interestingly, we conducted a linear analysis of the variables in both models. ITGB1 plays an important role in beta cell development and function, while some studies have found a positive effect of EIF4G1 on insulin secretion [46, 47]. Recent studies have shown that inactivation impairs insulin function [48], which supports the reliability of our results. This may be the mechanism behind pregnancy loss in PCOS. In the present study, we observed a significant correlation between ARSA Insulin and TSH expression, however, there is limited research on the relationship between ARSA and insulin which deserves further study.

Our study still has some limitations that require further study. Firstly, it was a retrospective study, the sample size was relatively small and the public database PCOS proteomics data was few. These findings need to be verified in future intervention studies. Secondly, although we obtained only three proteins with good predictive performance, the use of machine learning algorithms may miss some useful predictive factors that can’t be ignored. Thirdly, Numerous experiments are needed to verify how these proteins and pathways affect the receptive mechanism of the endometrium in PCOS patients.

## Conclusions

We conducted proteomic analysis of samples, screened DEPs and analyzed pathways related to PCOS and PCOS pregnancy loss. We further screened autophagy proteins and constructed a robust model. The model based on the ARSA, ITGB1 and GABARAPL2, demonstrated high predictive accuracy for identifying pregnancy loss in PCOS patients, thus providing a solid theoretical basis for future investigations.

### Supplementary Information

**Additional file 1.** Results of the Differentially Expressed Protein (DEP) analysis.

**Additional file 2.** The functional Enrichment Analysis of DEPs.

**Additional file 3.** 

## Data Availability

The analyzed data sets generated during the study are available from the corresponding author on reasonable request.

## Abbreviations

PCOS: Polycystic ovary syndrome
DEPs: Differentially expressed proteins
GO: Gene Ontology
KEGG: Kyoto Encyclopedia of Genes and Genomes
ROC: Receiver operating characteristic curve
DCA: Decision curve analysis
ET: Endometrial thickness
